# Engineering of Botulinum Neurotoxins for Biomedical Applications

**DOI:** 10.3390/toxins10060231

**Published:** 2018-06-06

**Authors:** Robert P. Webb

**Affiliations:** The Division of Molecular and Translational Sciences, United States Army Medical Research Institute for Infectious Diseases, Fort Detrick, MD 21702, USA; Robert.p.webb6.civ@mail.mil

**Keywords:** botulinum toxin, engineered toxin, recombinant botulinum toxin, drug delivery, chimeric BoNT, alternative binding domain, re-targeted BoNT

## Abstract

Botulinum neurotoxins (BoNTs) have been used as therapeutic agents in the clinical treatment of a wide array of neuromuscular and autonomic neuronal transmission disorders. These toxins contain three functional domains that mediate highly specific neuronal cell binding, internalization and cytosolic delivery of proteolytic enzymes that cleave proteins integral to the exocytosis of neurotransmitters. The exceptional cellular specificity, potency and persistence within the neuron that make BoNTs such effective toxins, also make them attractive models for derivatives that have modified properties that could potentially expand their therapeutic repertoire. Advances in molecular biology techniques and rapid DNA synthesis have allowed a wide variety of novel BoNTs with alternative functions to be assessed as potential new classes of therapeutic drugs. This review examines how the BoNTs have been engineered in an effort to produce new classes of therapeutic molecules to address a wide array of disorders.

## 1. Introduction

Botulinum neurotoxins (BoNTs) are a phylogenetically diverse group of AB protein toxins that disrupt the vesicular trafficking of neurotransmitters and result in a characteristic paralysis that may require extended supportive care. There are seven canonical serotypes of BoNT, denoted A-G, that are differentiated by their reactivity with polyclonal serum [1]. BoNTs are produced primarily by strains of *Clostridium botulinum* and in related *Clostridium* species including *C. butyricum* (BoNT/E) [2,3], *C. baratii* (BoNT/F) [4], and *C. argentinense* (BoNT/G) [5]. In addition to the seven serotypes, an astonishing number of subtypes with small to significant heterogeneity at the amino acid level have been characterized [6,7]. Additionally, chimeric toxins that appear to be evolutionary genetic rearrangements of existing serotypes have been reported in the last two decades [8,9,10]. Within the last two years alone there have been three novel new serotypes that were identified not from immunological profiling of clinical isolates, but from in silico data mining experiments of genomic databases [11,12]. BoNT/X was identified from a database search of *Clostridium botulinum* strain 111 [13], an isolate from an infant botulism case attributed to BoNT/B2 [14]. Two of these recent discoveries are “BoNT-like” genes characterized from non-*Clostridium* bacteria. A 2015 study reported the genome of *Weissella oryzae* SG25T, a Gram-positive bacterium isolated from fermented Japanese rice, was found to possess a BoNT-like gene [15]. Another BoNT-like gene was later discovered in *Enterococcus* sp. 3G1_DIV0629 [16]. There is no evidence of any native expression of these three novel BoNTs. 

The BoNTs all possess three individual functional domains that facilitate binding, internalization and toxin translocation into the cytosol of target cells. The light chain (LC) is a ~50 kDa Zn^2+^ dependent metalloprotease that represents the actual toxic domain of the holoprotein. The ~100 kDa heavy chain (HC) is functionally distinguished into the ~50 kDa translocation domain (HN) and the ~50 kDa receptor binding domain (RBD). BoNTs are produced as 150 kDa single chain (s.c.) proproteins that are enzymatically cleaved into a di-chain (d.c.) form in which the LC and HC remain linked by a single disulfide bond [17]. Preliminary binding of the RBD is achieved through a dual receptor binding mechanism. The initial interaction occurs between the toxin RBD and the abundant complex polysialo-gangliosides on the outer leaflet of the presynaptic membrane. This preliminary interaction is reinforced by secondary binding to specific receptor-class proteins, synaptotagmin (Syt) and synaptic vesicle glycoprotein 2 (SV2) in a serotype dependent fashion, [18,19]. The toxin is internalized into an early endocytotic vesicle in which the reduction in pH facilitates a state change in the HN, promoting the formation of an endosomal pore through which the LC can be extruded in the cytosol [20,21]. This process is facilitated by two key processes. The reduction of the disulfide bond is achieved by the thioredoxin-thioredoxin reductase enzyme which liberates LC from the HC [22]. The heat shock protein 90 (Hsp90) has been demonstrated to serve a molecular chaperone, re-folding the LC into its functional form [23]. Once in the cytosol, the LC cleaves synaptosomal *N*-ethylmaleimide-sensitive attachment protein receptors (SNAREs) in a serotype dependent fashion [24,25]. Serotypes /A, /E and /C cleave synaptosomal-associated protein 25 (SNAP-25); BoNT/B, /D, /F and /G all cleave vesicular associated membrane protein (VAMP), and BoNT/C cleaves both SNAP25 and syntaxin. BoNT cleavage of the SNARE proteins interrupts the vesicular trafficking of neurotransmitters that results in a classic, descending bilateral paralysis that if not addressed, can cause death from respiratory arrest. 

There are currently no US-licensed vaccines against botulism. Monovalent toxoids against serotypes A-E were produced from 1969 to 1971 by the Michigan department of health (MDPH) and subsequently formulated into a pentavalent BoNT toxoid (PBT) vaccine. Four individual lots of PBT were released for use in the US and administered under an investigational new drug (IND) license held by the Centers for Disease Control and Prevention (CDC) (IND 161) for at-risk workers and U.S. Army Office of Surgeon General (IND 3723) for use with military personnel at-risk during deployment [26,27]. However, the PBT vaccine exhibited declining potencies in the mid-1990s and by 2004 was found to elicit anti-PBT antibodies in less than 15% of the vaccinees [26,27]. Because of the diminished potency, the CDC announced it would no longer provide investigational PBT after 30 November 2011 [28]. However, IND 3723 remains active and the PBT could still be used to vaccinate military personnel. The majority of the subsequent BoNT prophylaxis efforts focused on the assessment of non-toxic, recombinant subunits to elicit protective immunity, particularly RBD-based vaccines [26,27,29]. A recombinant bivalent vaccine composed of the RBD from serotypes /A1 and /B1 (rBV A/B) was found to be highly immunogenic [30,31], well tolerated and has successfully completed a phase 2 clinical trial in 2015. Current treatment for BoNT intoxication consists of administration of antitoxin to reduce the duration and severity of the disease and if required, ventilatory supportive assistance. Infant botulism, in children under 1 year of age, can be treated with BabyBIG^®^, Botulism Immune Globulin Intravenous (Human) (BIG-IV). The antitoxin, developed by the California Department of Public Health (CDPH) and licensed in 2003, was derived from hyperimmune plasma donated from volunteers boosted with the PBT vaccine [32]. With the discontinuation of the PBT vaccine, the Joint Vaccine Acquisition Program (JVAP) and the Dynport Vaccine Company LLC. (DVC) coordinated with the CDPH to investigate assessing the rBV A/B as a potential replacement immunogen for boosting volunteer plasma donors for production of BIG-IV with promising results [33]. In 2010, HBAT (Botulism Antitoxin Heptavalent (A, B, C, D, E, F, G)) became the only botulism antitoxin available in the United States for naturally occurring non-infant botulism. The HBAT is a de-speciated scFv preparation that has been demonstrated to be effective in NHP models [34] and to be well tolerated and effective in human intoxications [35]. 

BoNTs represent one of the most potent protein toxins ever characterized with an estimated human oral lethal dose of 30 ng, 0.80 to 0.90 microgram (mcg) by inhalational route, and 0.09 to 0.15 mcg by the intravenous route [36]. While human botulism is caused exclusively by BoNT/A, /B, /E and /F, all seven serotypes have been demonstrated to be lethal in non-human primates [37]. The relative ease of production of natural and recombinant toxin preparations, the potency and duration of the BoNTs, the lack of any licensed prophylactic product combined with the potential need for long term supportive care have resulted in these toxins being categorized as Tier 1 select agents. However, the very properties that make BoNTs a formidable potential biological weapon, also make them a highly effective drug with an ever-expanding list of therapeutic applications in the treatment of spasticity disorders [38,39,40]. The potent, extended activity of BoNT in specific classes of neurons has made certain toxin serotypes effective drugs for the treatment of neurological disorders, and in 1998 BoNT/A was licensed by the Food and Drug Administration (FDA) for the treatment of strabismus and blepharospasms. Since then, BoNT/A and BoNT/B have been successfully employed to treat an ever-increasing variety of neuromuscular disorders and there are currently four FDA licensed BoNT products used in the US based on serotypes A (Botox^®^, Dysport^®^ and Xeomin^®^) and B (Myobloc^®^). 

The modular arrangement of the three functional toxin domains that facilitate specific receptor binding, cellular internalization and target proteolysis have made the BoNTs desirable molecules for engineering to produce novel proteins with new applications. This review summarizes engineered BoNTs designed for a wide variety of novel applications as well as elucidating new properties of the native toxins that may extend their list of current therapeutic applications. When available, details on the design and production of the recombinant proteins, the amounts used in the in vivo and in vitro assessments, the results and the author’s interpretations have been included for comparative analysis.

## 2. Recombinant Full Length Atoxic BoNT

The use of atoxic BoNT derivatives has been particularly successful in the development of therapeutic drugs. The ability to rapidly synthesize expression platform-specific open reading frames (ORFs) and assess individual neutralizing mutations has made the production of safe, non-toxic BoNT surrogates common in the development of new vaccine antigens. One of the earliest examples of an engineered, recombinant, full length attenuated BoNT protein was a 1997 report that documented the production of a BoNT/C1 with LC mutations H^229^G, E^230^T, and H^233^N [41]. The recombinant attenuated BoNT/C1 was expressed in *E. coli* and purified via an amino-terminal 6X polyhistidine (6XHis) affinity tag. The modified BoNT/C1 was found to be completely atoxic in mice at 10 micrograms (mcg) administered intraperitoneally (IP). The atoxic BoNT/C1 was also assessed by the moue phrenic nerve hemi-diaphragm (MPN) ex vivo assay. Briefly, this assay is performed by measuring generated electrical impulses across a mouse phrenic nerve in dissected mouse hemi diaphragm preparations maintained in Tyrode’s buffer [42]. Toxin is added to the buffer and the loss of the muscle “twitch” (paralysis) is quantified as a metric for determining toxin potency. The native BoNT/C at 10^−12^ M induces paralysis after 152 min but the atoxic recombinant BoNT/C1 displayed no physiological effects in the MPN assay at 10^−10^ M. Non-adjuvanted atoxic BoNT/C1 was administering at 2 mcg subcutaneously (SC) or 4 mcg intragastrically (IG) in mice at 0, 14, 28 and 42 days. The mice were challenged with 100 LD_50_ of BoNT/C1 three months after the last boost. Both the SC and IG dosage routes provided complete protected against the native toxin challenge. 

Webb reported the use of *Pichia pastoris* codon-optimized, full length open reading frames (ORFs) encoding BoNT/A1, /B1, /C1, and /E1. These catalytically inactive BoNTs (ciBoNTs) were engineered with neutralizing mutations in the conserved LC active site HEXXH motif in which histidine and glutamic acid moieties were substituted with non-reactive alanine residues (Table 1) [43,44]. The ciBoNT HPs were purified with a combination of ion exchange (IEC) and hydrophobic interaction chromatography (HIC) techniques, employing no affinity purification tags. The ciBoNTs were produced almost exclusively as s.c. proteins and were determined to be non-toxic in mice when administered IP at up to 50 mcg (ciBoNT/A1) and 25 mcg (ciBoNT/B1, ciBoNT/C1, and ciBoNT/E1). The ciBoNTs, adjuvanted with Allhydrogel, were comparatively assessed as vaccine candidates against the corresponding RBD subunit vaccine using a mouse single-shot potency bioassay. Briefly, mice are given a single dose of the antigen in an escalating dose schedule and challenged with 1000 MIPLD_50_ of either the parental toxin, or subtype toxin. The ciBoNT/A1 potency values against challenges of BoNT/A1, /A2 and /A3 were 18 ng, 132 ng and 144 ng, respectively. The RBD vaccine potencies against the same toxin challenges were 52 ng, 5.9 μg and 18 μg. The ciBoNT/B1 elicited similar results with potencies of 19 ng, 67 ng and 32 ng against challenges of BoNT/B1, /B2 and /B4. The BoNT/B RBD potencies against the same toxin challenge were 33 ng, 24 mcg and 77 mcg, respectively. The ciBoNT/C1 produced a potency of 15 ng against the parental /C1 toxin and 27 ng against the highly dissimilar /CD chimeric toxin. The /C1 RBD vaccine produced a potency of 101 ng against the parental toxin challenge but no potency against the /CD due to lack of survivors. The ciBoNT/E1 HP had a potency of 12 ng against a challenge of /E1, 4 ng against the highly similar /E3 and 22 ng against subtype /E4. The BoNT/E1 RBD vaccine elicited a potency of 1.13 μg against the parental toxin, 2.24 mcg against /E3 and >10 mcg against /E4. In an independent study, the ciBoNT/A1 and /B1 antigens were used to inoculate alpacas using a 100 mcg parental dose and 3 individual 50 mcg boosts at 3 weeks intervals [45]. Lymphocyte preparations were used to construct scFv libraries that identified a panel of high binding affinity camelid anti-BoNT heavy-chain-only Ab VH (VHH) antibodies that, when used as heterodimers, provided post-intoxication clearance in low toxin dose (10 LD_50_) challenges in mice.

Yang et al. described recombinant a BoNT/A1 with neutralizing mutations E^224^A/E^262^A produced in *E. coli* purified via a carboxy-terminal 6X His tag and dubbed DR BoNT/A (Table 1) [46]. The endopeptidase activity of DR BoNT/A was found to be negligible against SNAP-25 at 200 nM as determined by ELISA. DR BoNT/A was found to be non-toxic when administered to mice at 1 mcg delivered IP [38]. Fluorescently labeled DR BoNT/A was found to selectively bind and internalize in human SH-SY5Y cultured neuroblastoma (neuronal) cells, but not human rhabdomyosarcoma muscle (non-neuronal) cells. The authors concluded the recombinant protein displayed good native target specificity and so was a potential a drug delivery vehicle (DDV) [47]. In a 2013 follow up study, the interaction of DR BoNT/A in motor nerve terminals was more thoroughly characterized using in vitro and ex vivo assays [48]. DR BoNT/A was compared to native type /A toxin in the in vivo extensor digitorum longus (EDL) toe spread assay (TSA). Briefly, a small incision is made where the peroneal enters the EDL muscle and native /A toxin, or the DR BoNT/A surrogate is injected into the point of innervation and the incision is sutured. The effect of the toxin is scored by the TSA in which the mouse, lifted by tail peroneal muscles, reflexively spread the 2nd, the 3rd and the 4th toes, indicating normal peroneal nerve function. The reflexive toe spread is scored from 0 (normal) to 4 (maximum reduction in digit abduction) [49]. While only 3.2 picograms (pg) of BoNT/A toxin was sufficient to induce localized paralysis, 150 ng of DR BoNT/A elicited no observable paralytic effect. A similar ex vivo assessment of the EDL was conducted using EDL nerve-muscle preparation (NMP); similar to the MPN assay. Briefly, the EDL muscle containing the peroneal neve is excised, mounted in a buffer chamber and the electro-stimulated neural activity is measured to quantify motor neuron end plate currents (EPCs). Approximately 10 pM of native BoNT/A toxin was found to completely ablate evoked acetylcholine (ACh) neurotransmitter release from MNTs while 10 nM of DR BoNT/A had no inhibitory effect. The authors concluded the data describe the non-toxic effects of DrBoNT/A at the MNTs and reiterated its potential as a non-toxic surrogate. In a 2016 study, the endpoint toxicity of DR BoNT/A was determined using a range of 2.5–100 mcg per mice and the MIPLD_50_ was found to be 75 mcg [50]. In this same study, mice were inoculated intranasally (IN) with 5 mcg of DR BoNT/A at 0, 14 and 28 days. Vaccinations formulations comprised of: the atoxic protein alone, with mucosal adjuvants Vitamin-E TPGS (1.0%, *w*/*v*) or Forskolin (2.0%, *w*/*v*), or adsorbed onto poly(lactic-*co*-glycolic acid) (PLGA) microspheres,. The mice, in all the different IN formulations, were completely protected against a challenge of 1000 MIPLD_50_ of BoNT/A1. The authors note this is the first published report of a full length inactive holotoxin administered via inhalation route to elicit protective immunity against a substantial toxin challenge. 

A recombinant inactive BoNT/A1 with LC mutations R^363^A and Y^365^F, denoted BoNT/A^RYM^, produced in *C. botulinum* strain LNT01 and purified with a 6XHis tag (Table 1) [51]. The purified, recombinant BoNT/A^RYM^ injected at up to 1 mcg IP in mice, in either s.c. or d.c. form, elicited no symptoms of botulinum intoxication. Mice were vaccinated with 0.1, 1.0 or 10 mcg of the BoNT^RYM^ adjuvanted to Alhydrogel at 0, 14 and 28 days (adjuvant omitted for the final boost). The mice were challenged with the parental toxin at 7 days post-inoculation and 1 mcg was found to provide complete protection against 10,000 MIPLD_50_. Sera from surviving mice was found to neutralize 10 nM of BoNT/A in rat cortical neurons (CNs) as visualized by fluorescent microscopy using a SNAP-25 monoclonal antibody (Mab) that distinguishes the cleaved from of the SNARE. Band et al. purified a series of atoxic BoNT/A derivatives (BoNT/A1^*ad*^) with LC attenuation mutations E^224^A, Y^366^A and additional mutations K^438^H, K^440^Q, K^444^Q and K^871^N to address potential spurious cleavage from trypsin-like proteases present in the expression platform (Table 1) [52]. The *Spodoptera frugiperda* codon-optimized constructs were expressed in *Sf9*-baculovirus cells using a honey bee melatin (HBM) eukaryotic secretion signal and purified via a 10X His tag cleaved via an engineered tobacco etch virus (TEV) site. The recombinant BoNT/A1^*ad*^ was cleaved into the d.c. form via engineered internal TEV or enterokinase (EK) sites. The BoNT/A^*ad*^ toxicity was determined by IP administration in mice was found to have an LD_50_ of 8.9 mcg as a s.c. and 1.12 mcg in the d.c. form. [53]. Murine diaphragm preparations removed from mice 12 h post-injection IP with 1 mcg of BoNT/A^*ad*^ and incubated with a LC-specific MAb indicated the atoxic BoNT specifically binds neuromuscular junctions (NMJs) in vitro. A follow up study reported that cultural rat hippocampal neurons (HCNs) incubated with 15 nM BoNT/A^*ad*^ for 1 min and incubated with a BoNT/A LC specific antibody demonstrated the derived LC^*ad*^ was found to persist in the neuronal cytosol for up to 11 days. The authors stated the results indicated not only suggested binding and internalization, but endosomal escape of the cleaved LC^*ad*^ into the cytosol; again, suggesting the engineered BoNT/A could be utilized as a vehicle for delivery [54]. However, Cyto-012 BoNT/A, a recombinant BoNT/A with the same E^224^A, Y^366^A mutations, also produced in an *sf9* baculovirus expression platform was reported to have an intramuscular LD_50_ (IMLD_50_) of approximately 0.63 mcg as determined by the mouse DAS [55]. This suggested that, like the native toxin, the route of administration may be relevant when assessing recombinant atoxic BoNT derivative. The authors note that Cyto-12 possesses a pharmacologic profile like wt BoNT/A in terms of the onset and delay of the DAS response when used at higher concentration of 0.63 μg. The authors explain increased local uptake could result from the higher concentrations of Cyto-012 injected (relative to the native BoNT/A) increasing local neuronal uptake by mass action. They also hypothesize the localized uptake of Cyto-012 may be increased because it cleaves SNAP-25 at a slower rate than the wild type toxin (wt) BoNT/A and so displays diminished ability to disable SV2-mediated uptake of BoNT/A. Finally, the authors state that while the reduced activity of the Cyto-012 may make it useful in some clinical applications; its relatively narrow therapeutic range of concentration may limit its use as a drug delivery vehicle (DDV). The potential DDV limitations of the BoNT/A^*ad*^ was addressed by the follow up study on the design, production and assessment of a recombinant BoNT/C1^*ad*^ protein (Table 1) [56]. The BoNT/C1^*ad*^ consisted of a *S. frugiperda* codon-optimized ORF with an amino-terminal 10X His tag, BoNT/C1 with LC with mutations E^238^A, H^241^G and Y^383^ A, a hemagglutinin (HA) tag and three Strep tag (ST) repeats with engineered TEV sites after the His tag, in between the LC and HC and between the HA and Strep tags. The BoNT/C1^*ad*^ protein was produced and purified as described previously for the BoNT/A1^*ad*^. The MIPLD_50_ of BoNT/C1^*ad*^ was determined to be 5 mg/kg and was confirmed by two independent laboratories. The data suggests the introduction of an additional LC histidine mutation in the atoxic BoNT/C1 substantially decreased the residual toxicity. The authors state this BoNT/C-based DDV is 5 × 10^6^-fold less toxic than native BoNT/C1 toxin and that extrapolation from the mouse studies suggests that 56 mg would be a safe, high-end dose in an average 70 kg human. The authors further elaborate upon the choice to change to a serotype /C1-based DDV rather than further modification of the /A serotype model. The majority of the reported intoxications in the US are from serotype /A [57] and any therapeutic cargo in a serotype /A DDV might result in in intramolecular interaction and deactivation of the therapeutic. The use of a BoNT/C1^*ad*^ would permit inclusion of therapeutics to not only type A intoxication therapies, but to those caused by /B, /E and /F as well. The authors also cite the potential advantage that although the BoNT/C1 receptor has yet to be conclusively identified, the native toxin BoNT/C1 is able to enter neurons that were previously intoxicated with wt BoNT/A1 [58]. This suggests that BoNT/A and BoNT/C might employ different mechanisms of internalization. If accurate, this could facilitate a serotype /C1 DDV with BoNT/A1-inactivating therapeutic entry into the neuronal cytoplasm of BoNT/A1-intoxicated neurons. 

An *E. coli* optimized ORF consisting of an amino-terminal 6X His tag, 2 HA epitopes, BoNT/A1 with LC neutralizing mutations E^224^A, R^363^A, Y^366^F and a carboxy terminal ST, denoted M-BoNT/A1, was expressed in a prokaryotic expression platform, purified via the affinity tags (Table 1) [59]. An identical construct with additional RBD domain ganglioside binding pocket mutation W^1266^A, previously shown to impair binding/internalization of a recombinant RBD into cultured rat neuronal cells [60], was also produced. Both protein antigens were assessed to determine their ability to elicit protective immunity against a substantial challenge against the parental toxin, subtype /A2 or a cocktail of BoNT /A2 /A3 /A5 and /A6 subtypes. Mice were inoculated IP with 0.3 μg of each protein, adsorbed to Alhydrogel, at days 0 and 14 and challenged on day 26. Both protein antigens provided complete protection against a challenge of 10^5^ LD_50_ of BoNT/A2. The M-BoNT/A1^W^ protected against 10^6^ LD_50_ challenge of the parental toxin and against a 10^5^ LD_50_ challenge of the BoNT/A subtype cocktail. Recombinant RBD^W^ and LC-HN protein antigens elicited 70% and 90% protection against the same challenge level of the BoNT/A subtype cocktail. The authors noted the results of this study supported other investigations demonstrating the superior protective immunity elicited by the full length, enzymatically attenuated BoNT protein antigens compared to the individual RBD domain-based vaccines.

## 3. Recombinant Functional BoNTs

While a great deal of efforts has been dedicated to producing recombinant BoNT proteins with extremely low, or no detectable residual catalytic activity, there is a substantial need for well characterized functional BoNT reference materials. Such reagent grade toxins would help facilitate the development and validation of biological assays to assess therapeutic agents. The heterogeneity of commercial and locally produced lots of BoNT, combined with differing or incomplete characterization of these reagents, could result in deviations or conflicting data when testing identical therapeutic drugs. Even the highly characterized pharmaceutical grade BoNT/A preparations of Botox^®^, Dysport^®^ and Xeomin^®^ have different manufacturing processes that result in distinctive pharmacological differences that affect clinical activity and prevent efforts at cross-product dose equivalence [61]. While the primary focus of this review is focused on engineered BoNT derivatives, the efforts at producing laboratory grade functional BoNT reference material warrants recognition. The Establishment of Quality Assurance for the Detection of Biological Toxins (EQuATox) consortium, comprised of several different EU members (http://www.equatox.eu/), has produced recombinant, highly characterized functional BoNT/A1, /B1, /C1, /E1, and /F1 reference materials [62]. Codon-optimized ORFs for these five serotypes were expressed in an *E. coli* expression platform and purified using either carboxy-terminal 6XHis or ST affinity tags with engineered thrombin sites to facilitate cleavage to the d.c. form and to remove the affinity purification tags. The recombinant BoNT reference materials were analyzed by a wide variety of biophysical and immunological techniques and in vitro and in vivo assays [62,63,64] and found to be highly consistent with the native toxins.

Recombinant DNA technologies have also been shown to be useful in the characterization of functional BoNTs in *Clostridial* strains that produce two or more individual toxins. A bivalent strain reported from an infant botulism incidence in 2014, designated *C. botulinum* strain IBCA10-7060, was reported to express both a /B2 toxin and a novel new toxin denoted serotype H [9,10]. The nomenclature was based on the fact that the unknown BoNT was only weakly neutralized by the antibodies against currently known serotypes. Sequence analysis of the translated BoNT/H ORF indicated the LC had 80% homology with the BoNT/F5 LC, the HN region had 64% homology with /F1 and the RBD shared 84% homology with /A1 [65,66]. Furthermore, joint but independent studies from the University of Wisconsin and the CDC confirmed the novel BoNT was eliminated by existing serotype A antitoxins, including those that are constituents of multivalent therapeutic antitoxin products [66]. Collectively, this data suggests that BoNT/H would more accurately be described as an FA chimeric, rather than a novel new serotype. However, to more accurately assess the BoNT/FA toxin, two independent approaches were pursued to eliminate the BoNT/B component of the bivalent strain. To facilitate purification and characterization of BoNT/FA from the dual-toxin-producing strain CDC69016 (the CDC designation for the IBCA10-7060), BoNT/B2 was genetically eliminated in this strain by inactivation of the /B2 gene using the ClosTron mutagenesis system. The resultant strain was designated CDC69016/B2tox^_^ [67]. The purified /FA toxin was assessed and found to confirm many of the findings of the previous studies on the bivalent supernatants but also provided new information. Strain CDC69016 is genetically most closely related to Group I proteolytic *C. botulinum* strains but the /FA toxin is produced almost exclusively in the s.c. form. Trypsinization to the d.c. form was found to elicit a 38-fold increase in toxicity in the mouse toxicity bioassay. The d.c. form also displayed a 20-fold increase in toxicity as determined by VAMP-2 substrate cleavage in pluripotent stem cells (hiPSC) derived neurons. The highly purified toxin was also assessed by mass spectroscopy analysis where BoNT/FA cleaved VAMP-2 between ^54^L and ^55^E. These findings were independently verified [68]. The results of a mouse potency bioassay indicated the purified /FA had an activity of approximately 3.8 × 10^−7^ LD^50^/mg, roughly 5-fold lower than the activity of BoNT/A. However, the mice displayed a delayed time to death that is atypical of the observations when the assay is performed with BoNT/A. Neutralization studies were performed using 4 different polyclonal antibodies against BoNT/A1 in an in vitro cell-based assay using hiPSC-derived neurons. All four antibodies were found to neutralize the /FA toxin although at a 16-fold lower efficiency that against BoNT/A1. An in vivo mouse neutralization study using an equine BoNT/A1 complex showed protection against the /FA toxin was roughly 20 times lower than the protection observed against he /A1 toxin. Mouse neutralization studies using an /F1 antibody complex provided minimal protection. Finally, mice injected with 500 mLD_50_ of BoNT/FA with hBAT (0.125 mL per mouse) were 100% protected, suggesting that the hBAT is protective against BoNT/FA. 

A 2018 study detailed the production and characterization of a fully recombinant /FA toxin in *E. coli* [69]. A codon optimized /FA ORF was synthesized with an engineered BoNT/F1 (K^430^-L^444^) activation loop in place of the native BoNT/FA activation loop (S^429^-L^437^). The /F1 loop is larger by six amino acids and was found to facilitate Lys-C cleavage. The authors state that because BoNT the activation loops have poor sequence conservation and lie outside of structural domains, the F^1^ to F^5^ substitution is not likely to modify the recombinant /FA characteristics. Comparative neurotransmitter release of the modified recombinant BoNT/FA (mrBoNT/FA), native BoNT/A1 and BoNT/F1 were performed in rat embryonic spinal cord neurons (eSCN) and rat CNs by quantifying [^3^H]-glycine release. The rmBoNT/FA was found to be more potent than the native toxins in both assays (the ranking was BoNT/FA > A1 > F1) but the mrBoNT/FA-his was approximately three log units more potent than nBoNT/F1 in rat eSCN compared to approximately one log unit in cortical neurons. The authors speculate the significant difference in potency observed in the recombinant /FA and /F1 may be attributed to variations in receptor densities in these two cell types. Conversely, the mrBoNT/FA was found to be less potent than either BoNT/A1 or /F1 in both murine and rat PN assays. The authors note same variation in previous studies in which the BoNT/FA displayed higher potency than /A1 in cell culture assays, but lower in mouse bioassay [67,70]. The authors hypothesize that the differences observed between the in vitro and ex vivo assays might be attributed to the shorter time between toxin exposure and measurement in the Phrenic nerve assay (PNA) compared to the cell-based assay. They also postulated the slower enzyme kinetics of the BoNT/FA protease may cause the low potency inhibition of muscle contraction in the mouse PNA assay. The authors also suggested that intoxication of Peripheral motor neurons (PMNs) could differ from that of central spinal cord or cerebellar neurons. 

The production of recombinant functional BoNTs has also been shown to be useful in the characterization of the novel BoNT-like genes discovered by in silico searches, but not known to be produced by their individual host strain. The first published report of a non-*Clostridium* species was a 2015 study in which BoNT-like ORFs were identified in a recently sequenced *Weissella oryzae* SG25 genome [15]. While the sequence analysisindicated substantial *bont* gene homology, the local genome was atypical of most BoNT gene clusters, lacking the additional genes encoding for the neurotoxin accessory proteins. To ascertain the actual physical properties of this novel BoNT-like gene, *E. coli* codon optimized ORFs encoding the LC and RBD were expressed and purified via an amino terminal 6XHis tag [71]. The recombinant Wo-ORF-LC were found to cleave recombinant rat VAMP-2 at the Trp^89^-Trp^90^ peptide bond; a novel cleavage site compared to other BoNTs. The authors hypothesize that because the cleavage site is located within the juxtamembrane segment of VAMP-2, Wo-ORF1-LC would cleave the VAMP cytosolic domain and likely prevent SNARE complex assembly. The immunoreactivity of the recombinant LC and RBD was assessed by ELISA against the seven CDC standard BoNT antisera. Except for a weak cross-reaction with the anti-BoNT/C and the anti BoNT/D antisera, the recombinant LC and RBD displayed no-cross reactivity. The collective data from these studies strongly indicates a potentially novel new BoNT, which the authors suggested be termed BoNT/Wo. 

As with the *W. oryzae* preliminary analysis, *C. botulinum* strain 111 BoNT/X characterization was initially performed on recombinant /X LC. The novel /X LC which was shown to cleave VAMP-2 in rat brain detergent extracts (BDE) at a novel site between R^66^ and A^67^, unique only to this serotype [13]. Interestingly, the /X LC was also found to cleave purified, recombinant VAMP-4 and 5 as well as Ykt6 produced from 293T (human embryonic kidney) cells. This represents the first published report of a BoNT with the ability to cleave SNARES other than VAMP-1, 2 or 3. Due to safety concerns, a full length recombinant BoNT/X protein was not pursued but instead limited amounts of the holotoxin re-constituted from two recombinant non-toxic BoNT/X subunits using a sortase mediated ligation. The sortase enzyme is a transpeptidase that catalyzes the ligation of adjacent glycines to form a peptide bond and create a contiguous protein. Limited amounts of a recombinant BoNT/X LC-HN with carboxy terminal LPETGG and a RBD with an amino terminal free glycine were produced in *E. coli* and enzymatically ligated with sortase. The recombinant BoNT/X was able to enter rat CNs and cleave VAMP-2; indicating the RBD is capable of recognizing and binding mammalian receptors. Dot blot assays using antisera from all seven serotypes and BoNT/CD were non-reactive with the BoNT/X and confirmed it is a new serotype. Approximately 0.5 mcg of BoNT/X was required to elicit paralysis in the mouse DAS assay, indicating a much lower *potency* in vivo. The study also reported the production of an atoxic recombinant, full length BoNT/X protein with R^360^A,Y^363^F mutations, denoted BoNT/X_RY_. The neutralized BoNT/X_RY_ was shown to be non-toxic in mice at 30 μg IP and will be used to developed anti-sera against this unique serotype. The authors note that the actual potency of the toxin may be influenced by the sortase linking, the fact the HC and LC-HN were produced and folded separately, and the additional linker between the HN and HC in ligated toxins. 

In 2018, Brundt described the discovery of a BoNT gene cluster from *Enterococcus* sp. 3G1_DIV0629, putatively designated eBoNT/J, from an investigational analysis of the whole genome sequence (WGS) database at the National Center for Biotechnology Information (NCBI) [16]. Almost simultaneously, Zhang described the discovery and preliminary characterization of a BoNT-like gene, denoted BoNT/En, from a commensal strain of *Enterococcus faecium* strain IDI0629 isolated from cow feces [72]. As with the BoNT/X and /Wo, the preliminary characterization of this novel strain began with an assessment of SNARE target cleavage using a recombinant BoNT/En LC. Incubation with rat BDE indicated that the /En LC cleaved VAMP-2. Mass spectroscopy analysis of recombinant VAMP-2 cleavage indicated the /En LC cleaves VAMP-2 between A^67^ and D^68^. HEK293. Cell lysates transiently expressing a wide variety of SNARES indicted the /En LC cleaved VAMP-1, 2 and 3, and syntaxin 1 and 4; however, SNAP-23 and 25 were cleaved as well which was surprising as there was very minimal SNAP-25 cleavage observed in rat BDE. However, in vitro assessments indicated the /En LC only cleaved VAMP-2 with high efficiency. Less so than the syntaxin or SNAP-25 substrates. Recombinant BoNT/En LC-HN with an engineered thrombin site incubated in rat CNs for 12 h resulted in the efficient cleavage of both VAMP-2 and SNAP-25 substrates; the potency was increased when the cleaved d.c. form was used. The authors note the disparity in the efficiency of SNAP-25 cleavage in vivo and in vitro was also observed in studies performed with serotype /C [73]. Furthermore, mass spectroscopy analysis of the SNAP-25 cleavage products indicated the cleavage occurs between K^69^ and D^70^, a site unique to this serotype only. A recombinant BoNT/En toxin was produced, in limited amounts, using the sortase mediated ligation method described earlier. The ligated toxin showed only 1.25X SNARE cleavage activity in rat CNs over the unligated LC-HN and RBD constituents. When used in doses up to 1 mcg, the BoNT/En elicited no detectable paralysis in the DAS assay. However, substitution of a BoNT/A1 RBD increased entry in CNs and a 1:100 dilution of the chimeric /En LC-HN-A1-RBD cleaved more VAMP-2 and SNAP-25 than the unligated constituents. Furthermore, 1 ng of the chimeric ligated toxin consistently elicited paralysis in the DAS assay. These results suggest the BoNT/En may not have high affinity binding in the cell lines that were used in the study. As with the recombinant BoNT/X, the authors suggest the atypical recombinant proteins structure may have an impact on its native activity. Finally, the ligated BoNT/En was immunologically assessed in a dot blot assay described earlier but in additional the canonical antibodies used, rabbit polyclonal sera raised against the BoNT/X_RY._ There was no observed cross reactivity and the authors concluded that the BoNT/En is indeed a unique new serotype. 

## 4. Drug Delivery

The ability of the BoNT toxins to exclusively target and enter a specific set of neuronal host cells has made them an attractive choice as a vehicle to deliver payloads of therapeutic molecules. Functional LC-HN domains have been shown to retain their catalytic activity when ligated, or expressed as recombinant proteins, with a wide variety of different cell type/receptor specific targeting domains. A series of recombinant BoNT/D proteins with four individual amino terminal fusion cargo proteins: 25 kDa dihyrofolate reductase (DHFR), 27 kDa green fluorescent protein (GFP), 62 kDa firefly luciferase (LUC) or an active BoNT/A LC, were produced in *E. coli* and purified via a carboxy-terminal ST (Table 2) [74]. A recombinant s.c. BoNT/D (scBoNT/D) was produced in the same expression system and used as a positive control. The catalytic activity of the scBoNT/D fusion proteins was assessed by incubation with recombinant ^35^S-labelled synaptobrevin 2 and all were found comparable with the scBoNT/D. The s.c. form of the four recombinant fusion proteins were assessed in the mouse PNA with the scBoNT/D as a positive control. It should be noted the s.c. BoNT/D displayed approximately 10% of the activity of the d.c. form, or the native toxin. Since the fusion proteins were applied as s.c., toxicity was determined using the dose dependent response curve of the scBoNT/D and expressed as the percentage of the scBoNT/D toxicity. The DHFR had no effect on proteolytic activity whereas the LUC exhibited only 7% activity and the GFP was further attenuated. The LC/A had slightly higher activity attributed to the dual LC activity on the SNARES (/A LC cleaving SNAP-25); suggesting the cargo was enzymatically functional. The VAMP cleavage activity of the s.c. form of the fusion proteins was assessed in rat brain synaptosomes where DHFR or LC cargos had little effect, but a diminished activity was observed with the LUC or GFP fusions. The authors noted the reduced enzymatic activity observed for GFP-scBoNT/D in the MPN and synaptosomes could be compensated for by extending the incubation time. The presence of low levels of functional LUC and GFP were detected in the cytosol. This was the first report of a functional enzymatic cargo protein being delivered into the cytosol by a BoNT DDV.

O’Leary et al. described a recombinant protein, encoded by an *E. coli* codon optimized 480 bp core streptavidin (CS), a BoNT/B1 ORF with LC neutralizing mutations E^231^A and H^234^Y and a non-cleaved carboxy terminal 6XHis tag, dubbed BoTIM/B (Table 2) [75]. The recombinant protein was trypsinized to facilitate d.c. formation and chemically conjugated to a recombinant, biotinylated lentivirus encoding a GFPreporter gene (lentiGFP). The lentiGFP-CS-BoTIM/B vehicle-payload complex was found to specifically increase GFP expression in cultured rat SCNs up to 37% over the non-targeted lentiGFP. Similarly, in vivo studies performed by injecting rat tracheal ligaments with the lentiGFP-CS-BoTIM/B suggested higher efficiency of reporter gene transfer into neurons by CS-BoTIM/B-targeted lentiGFP compared with the non-targeted lentivirus. The CS-BoTIM/B was also conjugated to a separate biotinylated payload of MTR-S25. This is a SNAP-25 ORF with R^198^T, D^179K^ and M^182^T mutations which drastically reduce wild type cleavage by BoNT/A, /C and /E, respectively, allowing it to facilitate exocytosis in the presence of these toxins. Pre-incubation of rat SCNs with S25-BoTIM/B1 was found to increase the minimal toxin dose needed to view SNAP-25 truncation, as determined by Western blot analysis, from 10 pM to 125 pM for serotypes /A and /E and from 250 pM to 500 pM for /C1 by specific substrate competition. To confirm the delivered S25 did indeed protect synaptic activity, a whole-cell patch-clamp adaptation technique was used to record frequency and amplitude of spontaneous synaptic transmission in neurons. Significant synaptic transmission was clearly evident upon treatment with 200 pM of BoNT/A or 600 pM of BoNT/E, whereas untreated controls had no detectable activity. However, 200 pM of BoNT/C1 was unable to protect synaptic transmission in either control or pre-treated neurons. The lack of protection observed with BoNT/C was explained by the toxin’s ability to also cleave syntaxin, which was not protected by a cleavage resistant, analog as was SNAP-25. The CS-BoTIM/B was also assessed as a targeted drug carrier (TDC) vehicle in a separate study using a non-viral moiety by conjugation to a biotinylated liposome bearing a payload of BoNT/A LC or BoNT/A or /F protease inhibitors. [76]. In the MPN assay, biotinylated liposome containing functional BoNT/A LC conjugated to the BoTIM/B1 (CS-BoTIM/B-TDC-A) elicited a 90% reduction in nerve tension at 75 min. The non-targeted liposomes with the same payload took 308 min for equivalent muscle twitch reduction. Encapsulated liposome payloads of anti-BoNT/A high-affinity peptide inhibitor (2-mercapto-3-phenylpropionyl-RATKMLGSG) and BoNT/F inhibitor (residues 32–65 of VAMP-2 with G^58^d-cysteine) were added to the tissue bath at 8.3 and 7.6 μM, respectively, 30 min prior to the addition of the toxin. Approximately 100 pM of BoNT/A or 0.5 nM of BoNT/F reduced the 90% loss of tension by 53 and 30 min, respectively. Parallel control MPN assays performed with the encapsulated inhibitors, without a CS-BoTIM/B targeting moiety, displayed no significant delay in loss of tension. This study also included the comparison of the RBD alone as a TDC. Recombinant CS-HC ⁄B-TDC-A was found to be significantly slower than CS-BoTIM /B-TDC-A, taking 125 ± 9.6 min to induce 90% neuroparalysis.

## 5. Hybrid/Chimeric BoNTs

Chimerics, the product of the combination of reciprocal corresponding domains between different serotypes, has resulted the development of molecules with hybrid function that may be applied as novel therapeutics. It has also yielded useful information about the structure and function of the BoNTs themselves. A 2008 study described the production of two recombinant, chimeric proteins using *E. coli* codon optimized ORFs: BoNT/EA consisting of BoNT/E LC-HN a DI linker, BoNT/A RBD and a 16 amino acid tail containing 2 trypsin sites and a 6XHis tag and a BoNT/AE consisting of a BoNT/A LC-HN, a ELGGGGSEL linker, BoNT/E RBD, and an additional 19 exogenous residues including trypsin cleavage sites and a C-terminal 6XHis tag (Table 3) [77]. 

Both chimeras, proteolytically cleaved to d.c. form with trypsin, were found to retain the basic BoNT functions of receptor binding, endosome channel formation, and translocation of an active protease into the neuronal cytosol. However, the serotype specific properties were shuffled to produce novel proteins with modified functions. In the MPN assay, there was little difference in the time to paralysis between the /EA and the /E. However, both were more rapid than times observed with the /A. The /AE chimeric appeared to be the least potent and evoked the least delay in loss of muscle twitch. In vitro toxicity of the /AE and /EA, as determined by IP administration in mice, was found to be 0.3 × 10^7^ and 0.7 × 10^7^ MIPLD_50_, respectively, lower potencies than either parental toxin (BoNT/A 30 × 10^7^, BoNT/E 10 × 10^7^). The authors suggest that the more rapid paralysis is a function of the LC and/or HN region of BoNT/E and is not influenced by RBD binding. The potential effect of endosomal acidification on LC translocation rate was further investigated by pretreating mouse PCN cultures with vesicular H^+^ ATPase inhibitors. These compounds slightly raise the acidic pH of the endosomal lumen, which may quantify the role of acidification in the intoxication pathway. The rate of intoxication, determined by SNAP-25 cleavage, from highest to lowest was /E, /EA, /A with /AE substantially lower than the other three. The study was repeated in superior cervical ganglion neurons (SCGNs) which yielded largely the same results. The authors attributed the more rapid endosomal translocation of /E LC-HN to the differences in toxicity. However, in CGNs the seemingly slower acting /AE, as well as /A, maintained the ratios of cleaved to intact SNAP-25 much longer than the /EA and /E toxins which display more rapid recovery of intact SNAP-25. In the last study in this report, a DAS assay found the most rapid onset with /E (3 h), followed by /EA (5 h) and 12 h for /A. However, full muscle recovery was observed more quickly for the /EA and /E, most likely owing to the instability of the E LC within the cytosol. Interestingly, the /AE chimera recovery took up to 37 days, not only outlasting all of the other toxins tested, but nearly equivalent to that of clinically used type A complex. The study detected no major differences in vitro for SNAP-25 proteolysis by /EA, BoNT/A, and /AE, indicating their LCs do not influence either their speed of action in cultured neurons or the onset of neuromuscular paralysis. The authors suggest the rapid LC endosomal translocation of the /EA might explain its unique properties. 

The /EA was investigated in an independent follow up study to determine the chimera’s novel properties ability to influence anti-nociceptive signaling pathways as a potential therapeutic drug for chronic pain [78]. The BoNT/EA at 100 nM was able to block the release of the capsaicin-invoked proinflammatory calcitonin gene-related peptide (CRGP) in both cultured rat trigeminal neurons (TGNs) and in rat brain stem slices. Neither of the chimeric parental toxins, BoNT/A and /E, had any significant effect. The authors attribute the chimera’s success to two individual features. First, the abundance of the SV2C receptor protein isoform, the prime acceptor for /A, on rat TGNs. Secondly, the ability of the BoNT/E LC to cleave the 26 C-terminal amino acid of SNAP-25, rather than the 9 residues cleaved by serotype /A. This would potentially reducie the requisite high affinity binding for synataxin in the formation of the SNARE complex. 

The unique properties of recombinant BoNT/A and /B chimeras were elucidated in a series of in vivo and in vitro bioassays [79]. These hybrids consisted of an *E. coli* codon optimized BoNT /AB chimera consisting of /A LC-HN, an ELGGGGSEL amino acid linker and a /B RBD and the inverse /BA chimera consisting of the /B LC-HN, amino acid DI linker, and an /A RBD. Both constructs possessed an engineered thrombin site between the LC-HN and a carboxy-terminal 6XHis tag (Table 3). The BoNT/AB and /BA chimeras were shown to bind to their cognate receptor proteins, SV2C and SyntII, respectively, in pull down assays. They were also demonstrated to cleave their cognate SNAREs, SNAP-25 and VAMP2; indicating the individual domains are functional with serotype specificity. Both chimera were roughly equivalent in their ability to induce paralysis in the MPN assay as their parental (LC-HN dictated) toxins; suggesting delivery into cytosol of the motor neurons. However, the chimeras also displayed unique properties derived from the domain recombination. The /BA toxicity in mouse CGN was 6 × 10^8^ mLD_50_/mg, twice the toxicity of BoNT/A (3 × 10^8^ mLD_50_/mg) and 20-fold greater than the /AB (0.3 × 10^8^ mLD_50_/mg). However, the /AB was found to cleave significantly more SNAP-25 than the parental /A toxin in mouse SCNs and elicited a longer lasting paralysis as assessed in the mouse DAS assay than even BoNT/A. A feature also observed in the /EA hybrid toxin described earlier [78]. An independent study was performed using a similar chimera, BoNT/AABB (capital letters denoting BoNT LC, HN, HC_N_, HC_C_ domain) produced in *E. coli*, purified via a carboxy-terminal ST and converted into the d.c. form using an engineered thrombin site (Table 3) [80]. A recombinant BoNT/A, denoted AAAA, was produced in the same expression platform and used as a control in these studies. The d.c. /AABB chimera was assessed in the MPN assay and displayed approximately 8.4-fold greater potency than the AAAA control in a dose-dependent fashion in the picomolar range. However, an in vivo model employing the murine running wheel assay (MRWA), found the AABB and AAAA both displayed equivalent potency and duration. The author noted that differences in the results between these two studies might be attributed to the difference in the genetic constructs, the individual methodologies and disparities in the information provided by pharmacological vs pharmacokinetic analyses and warrants further investigation and analysis. 

Pellett described the production of recombinant chimeric BoNTs composed of the individual domains of serotypes /A1 and /A3 which provided insight into the roles of the LC and HC in both the potency and duration of intoxications [81]. A chimeric A1LC/A3HC and the inverse A3LC/A1HC were inserted into *Clostridial* expression vectors and mobilized into the non-toxicogenic *C. botulinum* Hall A-hyper/tox- strain for expression (Table 3). The recombinant proteins were purified by mixed column chromatography, without the use any affinity tags and contained no additional amino acid epitope tags in an effort to maintain native structural integrity. Toxin potency studies in mice indicated the hybrids had roughly equivalent potency with the corresponding HC donor toxin; /A1 (1.2 × 10^8^), /A3A1 (8.3 × 10^7^), /A3 (5.9 × 10^7^) and /A1A3 (3 × 10^7^). However, the physiological reactions towards the chimeric toxins corresponded with the parental LC contribution. Mice injected with /A1 tend to display greater spasticity prior to death than observed with /A3 intoxication and the physiological reactions to the hybrid chimeras were more consistent with their LC parent toxin. Potencies were determined in hiPSC-derived neurons exposed to the toxins for 48 h. Western blot analysis of SNAP-25 cleavage by was performed by densitometry scanning techniques to produce calculated EC_50_ values. BoNT /A1 and /A3 had EC_50_ values of 0.34 and 4.9 where the chimerics produced values in between the parental toxins (/A1A3 is 0.7 and /A3A1 is 1.2). The authors interpret this as indicating the LC and HC might both play a role in reducing the /A3 potency in this model. Potency comparisons in cultured primary rat SCNs indicated that the /A1 and /A1A3 had similar durations of ~10 months where the /A3 and /A3A1 displayed an abbreviated effect of 5 months, indicating the duration of paralysis might be influenced by the LC contribution. This was supported by a comparative in vivo study in which a DAS assay with /A1 and /A1A3 exhibiting similar recoveries at about 12 days post injection where the mice injected with /A3A1 or /A3 exhibited full recovery at about 5 days; again, suggesting the duration is influenced by the LC. 

## 6. BoNTs with Modified Target Specificity

Modification of the SNARE substrate specificity or cleavage ability (potency) is an attractive target as it may change the therapeutic index or application of current BoNT based drugs. A 2011 study designed to differentiate the specific roles of SNAP-25 and syntaxin in BoNT/C1 intoxications described the identification of two BoNT/C1 LC mutants demonstrated to ablate SNAP-25 proteolysis [82]. Recombinant BoNT/C1 LC mutations L^200^W, M^221^W, I^226^W (denoted BoNT/C α-3W) and S^51^T/, R^52^N, N^53^P (denoted BoNT/C α-51) were unable to cleave SNAP-25 in extracts from cultured rat HCNs as determined by Western blot analysis. This work was extended in an independent study in which recombinant full length BoNT/C1 bearing these light chain mutations were used to further investigate the individual contribution of SNAP-25 and syntaxin cleavage of BoNT/C in vivo [83]. BoNT/C α-51, BoNT/C α-3W and a wild-type BoNT/C1 (wt-BoNT/C1) were produced in an *E. coli* expression platform and purified via a ST. In cultured rat CGNs, the two mutants cleaved syntaxin 1A/1B with roughly 10-fold less activity than the wt-BoNT/C, as determined by Western blot analysis. However, the mutants also displayed SNAP-25 cleavage in the CGNs, but at 50-fold (BoNT/C α-3W) and 100-fold (BoNT/C α-51) lower activity compared to the wt-BoNT/C1. The three BoNT/C1 isoforms were also assessed in CGNs by fluorescence microscopy using an antibody specific to BoNT/A1 primary SNAP-25 cleavage products. The wt-BoNT/C1 elicited a similar pattern to the /A1 and the mutants displayed staining indicative of reduced activity, reflecting the rates observed in Western blot analysis. Cytotoxicity assessments determined by staining of cellular neurofilament 200 (NF-200) indicated both the wild type toxin and BoNT/C α-51 caused substantial loss of structural integrity. However, there was no significant effect from BoNT/C α-3W. A mouse potency bioassay indicated the BoNT/C α-3W and BoNT/C α-51 displayed approximately 200 (LD_50_ = 150 ng/kg) and 1000 (LD_50_ = 750 ng/kg) fold lower toxicity than the wild type toxin (0.75 ng/kg) and the BoNT/C α-51 displayed a slower progression and longer time to death. The authors state the results suggest the potency of the BoNT/C1 isoforms may be related to their limited ability to cleave SNAP-25. The duration of the toxins was assessed in a DAS assay, using one calculated LD_50._ The BoNT/C α-3W displayed a rapid onset followed by a full recovery in 48 h while the BoNT/C α-51 duration exceeded even the wild type toxin. However, ex vivo electrophysiological analysis of the evoked junction potentials (EJP) in mouse soleus muscle to quantify accurate neurotransmitter release at the NMJ, indicated BoNT/C α-3W induced full paralysis 24 h post injection, but rapid recovery and total restoration of function in two weeks. The wild type toxin and BoNT/C α-51 displayed complete loss of neurotransmission for one week and incomplete recovery at up to 5 weeks; which the authors note the mutant duration does not positively correlate with the potency results. Syntaxin and SNAP-25 specific staining of the soleus muscles revealed that BoNT/C α-51 elicits initial SNAP-25 cleavage but a sustained loss of the majority of syntaxin 1B. Collectively the data suggest that while SNAP-25 cleavage is required for the complete loss of transmission, syntaxin cleavage may contribute to recovery of function and duration of paralysis. The authors conclude that the study shows relatively minor changes introduced to create the mutant forms can significantly impact biological activity and further investigation could yield to a novel new pharmaceutical.

Tao reported the production of an engineered BoNT/B designed to enhance binding to the human synaptotagmin 2 (h-Syt II) in an effort to try and increase the therapeutic index of the toxin [84]. An in silico screening based on the interactions between BoNT/B and murine synaptotagmin II from crystallography studies suggested RBD mutations E^1191^M/S^1199^Y could enhance binding to h–Syt II. This theory was supported by the expression and binding assessment of recombinant BoNT/B RBD candidates generated in the study. A synthetic ORF encoding an *E. coli* optimized BoNT/B containing the E^1191^M/S^1199^Y mutations, denoted BoNT/B_MY_, with a carboxy terminal 6XHis was constructed. A native BoNT/B, generated by site-directed mutagenesis (SDM) of BoNT/B_MY_ to restore the wild type amino acid sequence, was also produced as a positive control. The BoNT/B_MY_ was assessed in humanized neurons created by knock down (KD) of endogenous Syt I and expression of full-length h-Syt II in cultured rat CNs via lentiviral transduction. BoNT/B_MY_ was found to increase VAMP-2 cleavage by Western blot analysis and by using whole-cell patch-clamp techniques. The maximum inhibitory concentration (IC_50_) was determined to be ~11-fold lower than recombinant wild type BoNT/B; suggesting the modified BoNT/B might display greater binding affinity in human cells. An independent study, also designed to increase the potency of BoNT/B by increasing its specificity for the VAMP-2 substrate, was performed using comparative structural analysis studies between the BoNT/B and tetanus toxin (TeNT) LC domains [85]. An engineered, recombinant BoNT/B LC with a LC/T S’ pocket residue, S^201^P mutation was reported to enhance VAMP-2 cleavage 10X as determined by Western blot analysis of murine N2a cell lysates. In an independent follow up study, full length, recombinant BoNT/B1 and BoNT/B1^S201P^ were produced in *E. coli*, purified by a combination of SCE and HIC methods sans affinity tags and cleaved to the d.c. form. As with the BoNT/C1 mutants described previously in this section [84], the individual domain mutations were assessed in a full length recombinant version of the protein and subjected to a much more extensive functional analysis [86]. The BoNT/B1^S201P^ was found to have a higher catalytic activity than either the rBoNT/B or commercial preparation of the native toxin in cell free cleavage assays of VAMP-1 and VAMP-2. However, the BoNT/B1^S201P^ and rBoNT/B displayed equal potencies in vivo as determined by inhibition of release of neurotransmitter by [^3^H]-glycine in mouse SCNs and glutamate release in cultured rat CNs. The BoNT/B1, rBoNT/B1 or rBoNT/B1S^201P^ displayed no differences in the time to paralysis in the ex vivo assays of the MNP, at 50 pM, or the mouse smooth muscle bladder strip at 1 nm. Finally, the ED_50_ in the in vivo DAS assay (the dose needed to attain a score of 2 of a possible 4 based on rear limb paralysis) was 1.3 pg for rBoNT/B1 and rBoNT/B1^S201P^ and 1.0 pg for native BoNT/B1. A maximum score of 4 required 3.4 pg for BoNT/B1, and 3.5 and 5.2 pg/mouse for rBoNT/B1 and rBoNT/B1^S201P^, respectively. The detailed analysis of the rBoNT/B1^S201P^ indicated the increased substrate cleavage observed in cell free lysates, was not apparent in the ex vivo and in vivo assays. It should also be noted the native commercial toxin did display higher levels of toxicity and potency than the recombinant form in some of the studies. The authors note that prior to their study, the S^201^P mutation had only been verified in cell-free cleavage assays using VAMP-2 as substrate and their data indicates the potency of BoNT/B1^S201P^ was not significantly greater than the wild type BoNT/B1 toxins in any of their in vitro, ex vivo or in vivo assessments. They suggest that the SNARE cleavage rate does not determine overall potency and does not seem to be the rate-limiting step in the onset of action.

## 7. Alternative Receptor Binding Domains

The modular design of the BoNTs can be utilized to engineer alternative receptor binding domains that re-direct an active LC-HN to alternative target cells for a wide variety of potential applications. Early efforts at combining functional LC-HN domains to alternative targeting moieties were accomplished primarily by chemical conjugation methods. A BoNT/A LC-HN generated by trypsinization and subsequent thiolozation was chemically conjugated to a neurotrophic, monomeric 13 kDa murine nerve growth factor (NGF) and shown to be potently active in inhibiting neurotransmitter release from PC-12 cells (Table 4) [87]. 

The conjugated BoNT/LC-HN-NGF was shown to bind PC-12 cells that express a tropomyosin receptor kinase A (Trk) receptor, known to bind NGF. The BoNT/A LC-HN/NGF was found to inhibit tritiated noradrenaline [H^3^-NA] release and cleave SNAP-25 as assessed by Western blot analysis; indicating functional entry of the protein into the cellular cytosol. Similarly prepared BoNT/A LC-HN was chemically conjugated to a of 36 kDa homodimeric *Triticum vulgaris* wheat germ agglutinin (WGA) domain using SPDP (succinimidyl 3-(2-pyridyldithio) propionate) (Table 4) [88]. The BoNT/A LC-HN/WGA displayed a reduced IC_50_ in cultured eSCN when compared to BoNT/A, suggesting a difference in cell binding specificity. However, the conjugated protein was found to cause a significant dose-dependent decrease in insulin in hamster pancreatic B-cell line HIT-T15; a cell line known to be resistant to BoNT/A. A conjugate composed of BoNT/A1 LC-HN and a 30 kDa *Erythrina cristagalli* lecithin (ECL) was designed specifically for the ligand’s demonstrated ability to selectively targeting nociceptive afferents [89]. The rationale for the design was to use the conjugate as a proof of concept for a new class novel analgesic drugs. The BoNT/A1 LC-HN/ECL was non-toxic at 50 mcg administered IP in mice and the conjugate was found to inhibit release of the nociceptive transmitter substance P in rat cultured embryonic dorsal root ganglia (eDRG) neurons; an in vitro system representative of nociceptive afferents. In a different approach to linking heterologous functional protein subunits, Darios reported using self-assembling constituents of the SNARE complex to conjugate individual proteins in a process called “protein stapling” [90]. Recombinant protein fusions representing two individual components for the stapling technique were: (1) A BoNT/A LC, a thrombin cleavage site, a BoNT/A HN, a rat SNAP25B; and (2) rat synaptobrevin fragment (amino acids 25–84), and a BoNT/A RBD were both purified with a GST affinity tag (Table 4). The two recombinant protein chimeras were conjugated by admixing with a synthetic syntaxin-3 (amino acids 200–250) to mimic the neural SNARE complex and assemble the fusion proteins into functional, recombinant BoNT, denoted LcTd-SNARE-Rbd. The LcTd-SNARE-Rbd was found to successfully bind and be internalized into mouse HC neurons. Immunoblotting of the HC cell lysates with a SNAP-25 specific antibody indicated successful cleavage of the SNARE target protein. The assembled protein was assessed in an ex vivo assay in which mouse hind paw preparations (containing the flexor digitorum brevis) were incubated in a buffered bath containing 1 nm of the stapled protein or it constituents. The miniature end-plate potentials (MEPPs) were then assessed by patch clamp methodology. They noted a decrease in spontaneous exocytosis to 30% as compared to the controls treated with the individual, LcTd-SNAP25 and synaptobrevin-Rbd components. The LcTd-SNARE-Rbd also elicited a 50% paralysis in the MPN assay at 72 min at 90 pM; no control MPN assessments were reported in the study. Finally, the impact of the LcTd-SNARE-Rbd on organotypic slices of the suprachiasmatic nucleus (SC) region, the primary area of Circadian rhythm (CR) regulation were assessed. The samples were obtained from neonatal mice expressing a Per2-luciferase fusion protein (Per-2 is a key CR regulatory protein). Bioluminecsent quantification of the brain slices treated with approximately 6 nM of the stapled protein caused a rapid onset and persistent (5 days) disruption of Circadian pattern gene expression. A 2011 follow up study reinforced the initial assessments of the LcTd-SNARE-Rbd, now referred to as BiTox, but presented data from additional studies [91]. To determine if direct injection BiTox into the brain exhibits detectable BoNT activity, rat visual cortex regions were injected with either BiTox or a non-reactive control. Two day post-injection localized brain tissue, assessed by Western blot analysis, indicated SNAP-25 cleavage. Finally, the mouse toxin bioassay indicated doses from 20 to 200 ng/kg administered IP resulted in no symptoms of toxicity. The authors note the unusual characteristics of BiTox which efficiently influence the CNS, but does not seem to elicit neuromuscular paralysis. Subsequent unpublished results indicated BoTox was lethal in the 100–500 ng range. The authors reason the unusual properties may be the result of the increased size and unusual architecture of the stapled protein, which my prevent entry in to the NMJ. However, they also postulate that these same characteristics might find use in applications that benefit from a reversible silencing of only CNS neurons.

Ferrari et al. described the use of the protein stapling technique to assemble a re-targeted Clostridial chimera consisting of the functional BoNT/A LC-HN and a *C. tetani* RBD (Table 4) [92]. The ALC-HN/CtRBD was added to mouse HC neurons where it was found to cleave SNAP-25 in the low nanomolar range, as ascertained by Western blot analysis. The chimera was applied to slices from the mouse SC region and assessed by quantifying activity. At 5 nM, BiTox elicited a significant inhibition of normal CR patterns; potentially verifying its ability to affect native neuronal pathways in situ. However, the ALC-HN/CtRBD chimera was estimated to be 11,000 times less effective than the native BoNT/A in the mouse MPN assay. Approximately 500 ng of the ALC-HN/CtRBD administered IV or IM in mice elicited no signs of toxicity. The authors attributed this lack of toxicity to the larger size of the stapled chimera, a theory supported by the fact that a stapled BoNT/A of similar size and structure (BiTox), displayed similar properties [91]. However, the clostridial chimera successfully attenuated visual cortex stimulation in mice, suggesting the chimera could exert influence in a small, defined region of the brain. Finally, the ALC-HN/CtRBD was found to significantly reduce mechanical hypersensitivity in a Complete Freund’s Adjuvant (CFA) inflammation-induced hypersensitivity pain model in rats for up to 11 days. Collectively, the results suggest the ALC-HN/CtRBD does not readily elicit paralysis yet can be successfully employed to influence discreet areas of the CNS without systemic toxicity. 

A 2017 study documented preliminary efforts at synthesizing a modular delivery vehicle targeting cell surface specific receptors by inclusion of immunospecific conjugate molecules with the goal of influencing the tropomyosin kinase A (TrkA) mediated nociceptive pathway [93]. A recombinant molecule consisting a BoNT/A LC-HN with an engineered thrombin cleavage site, a linker consisting of 10 GS amino residues, and a *Staphylococcus aureus* domain B composed of five highly modular immunoglobulin G (IgG) interactive domains (A−E) bearing specific mutations to prevent hindrance of target binding, denoted LC-HN/A-SpA-B_mut_, was expressed in *E. coli* (Table 4). The vehicle was purified via GST affinity tag and conjugated to two different recombinant, receptor-specific ligands. The LC-HN/A-SpA-B_mut_ was coupled to an anti-tropomyosin kinase A (TrkA) IgG and the conjugated product was found to facilitate binding to NGF treated PC-12 cells and cleave SNAP-25 in a dose dependent fashion. The vehicle without the conjugated IgG did not promote entry. A more complex ligand consisting an *S. frugiperda*-optimized ORF encoding a full length human β nerve growth factor (β-NGF), residues 102−324 of rabbit Ig γ chain C region, a thrombin cleavage site and a 6XHis affinity tag was expressed in *Sf9* insect cells. The rFc-βNGF-(His)6 ligand conjugated to the LC-HN/A-SpA-B_mut_ carrier produced significantly greater SNAP-25 cleavage than equivalent concentrations of the uncoupled control constituents in PC-12 cells but was estimated to be proportionally less efficient that rFc-βNGF in facilitating transport. The protein stapling technique was used to add a variety of different alternative targeting domains including; epidermal growth factor (EGF), tumor necrosis factor α (TNFα), ciliary neurotrophic factor (CNTF); and neuropeptides: dermorphine (Dermo), dynorphin 17 (Dyn17), corticotropin-releasing hormone (CRH), substance P (SP), and somatostatin to a BoNT/A LCHN with the goal of targeting neuroendocrine tumor (NET) cells [94]. The constructs were assessed against five NET cell lines (PC-12, insulinoma Min6, neuroblastomas SH-SY5Y, Neuro2A, and pituitary AtT-20). The stapled EGF, CNTF, and CRH ligands were most effective in blocking endocrine secretory release. However, only the fully recombinant fusion proteins (not produced by protein stapling and without the SNARE components) with EGF and CNTF RBDs were as active as the stapled proteins in terms of SNAP-25 cleavage. Their findings indicate re-directed BoNT proteases could be designed to accurately targeted NET cells and ameliorate hypersecretion of hormones that result in endocrine disorders. Somm described the production of SXN101742 synthesized using an *E. coli* optimized BoNT/D LC, a Factor Xa protease activation site, a growth hormone releasing hormone (GHRH) peptide ligand domain (aa residues 1–40), a spacer a BoNT/D HN and a 6XHis affinity tag expressed in *E. coli* (Table 4) [95]. The SXN101742 protein was assessed as targeted secretion inhibitor (TSI) directed to somatotrophs to potentially reduce GH secretory granule exocytosis in an effort to treat pituitary GH hypersecretion disorders such as acromegaly. SXN101742 was assessed in vitro in rat GH3 pituitary tumor cells expressing a rat GHRH receptor (GH3-rGHRH-R). It was also examined in vivo by administering incremental doses of 0.1, 0.3, and 1 mg/kg by tail IV injection in rats. SXN101742 displayed a dose-depend, and almost complete, depletion of VAMP2 in GH3-rGHRH-R cells. Rats injected with SXN101742 exhibited a 25% hypertrophy in the pituitary glands. They also exhibited significant reductions in the GH release regulated proteins hepatic Igf1, acid-labile subunit (Igfals) and IGF-binding protein-3 (Igfbp3) proteins. Finally, the rats also displayed marginally reduced, transient delays in developmental body growth. The same recombinant SXN101742 TSI, denoted as qGHRH-LHN/D, was assessed in independent in study to ascertain if two CR factors associated with the Somm study would have any impact on the preliminary results. These are (1) the characteristic GH pulsatile ultradium rhythm (recurrent period or cycle repeated throughout a 24-h day) influenced secretion and (2) the short GH 14 min half-life [96]. Automated blood sampling (ABS) was taken every 10 min for 24 h from cannulated rats administered a single bolus of 0.3 mg/kg qGHRH-LHN/D as well as in a dose dependent study when the TSI was administered at 0.03, 0.1, or 0.3 mg/kg. The ABS results supported the original study findings on the effect of qGHRH-LHN/D on GH secretion from pituitary somatotrophs. However, the authors caution there is no valid animal model for acromegaly, there are technical limitations in these studies and while the results are promising, a more thoroughly engineered TSI may be required to specifically target and effectively treat pituitary hypersecretion disorders. Garcia reported on the comparison of the qGHRH-LHN/D activity in human cell lines derived from 25 acromegaly patients and 47 patients with nonfunctioning pituitary adenoma [97]. The qGHRH-LHN/D was shown to cleave GFP VAMP-2/3 in lysed pituitary adenomas as determined by the loss of immunofluorescence in Western blot analysis. However the chimera was unable to inhibit GH secretion or induce VAMP-2/3 cleavage in cultured somatotroph adenomas incubated with 0.3 μM of the TSI for 18 h. The authors noted this was contradictory to the in vivo animal studies described previously. The authors further cited other potential issues that may have led to the different results. First of all, there were several technical and experimental limitations of the study including the concentrations of the TSI used. They also cite differencing roles of VAMP in rat vs human pituitary GH secretion. Finally, they question if the qGHRH-LHN/D was even able to enter intact human adenoma cells or cleave VAMP. 

## 8. Conclusions

This review has detailed a wide variety of engineered BoNTs that have potential applications that include: non-functional toxin surrogates for vaccine use, antibody development and trafficking studies, applications in several different models of drug delivery, as TSIs designed to be used in nociceptive pathways for pain management and hypersecretion disorders and BoNT chimerics that have been found to have potentially new clinical applications as well as revealing new information about the native toxin domains used to create these hybrids. The most mature application of this technology is appears to be the application of atoxic BoNT derivatives to elicit protective immunity or generate antibodies. These recombinant atoxic BoNT derivates have been successfully produced in *E. coli*, *C. botulinum*, yeast and insect cell culture-based expression systems [43,44,51,52]. The catalytically inert BoNTs have been demonstrated to have significantly reduced toxicity (Table 1) that is far above the minimal therapeutic doses required as effective vaccines. These engineered BoNT derivatives have also been demonstrated to elicit greater protective immunity than the individual subunit vaccines, particularly against challenges with dissimilar subtypes [43,44,59]. This is most likely attributed to several different factors. First of all, the presence of additional epitopes on the inclusive domains in the holoprotein vs the individual subunit antigen. Secondly, the recombinant holoproteins may be more structurally accurate than the individual recombinant domains. This could produce more structurally accurate epitopes, including interdomain epitopes, not found on the subunit antigens. The ciBoNTs have also been successfully applied as safe, non-toxic surrogates in generating an antibody response for isolation of antibodies in camelids [45]. 

The recombinant functional BoNTs have potential as useful research tools. Contradictory results when generating and assessing identical novel new inhibitors, antitoxins and vaccines in different laboratories can sometimes be attributed to different toxin preparations. Producing botulinum toxins in house presents several difficult, and expensive challenges. These include: obtaining the correct strain of *C. botulinum*, establishing secure and regulatory compliant laboratory space, culturing an aerobic bacterium under conditions conducive to elicit toxin production, purifying the toxin, and accurately assessing the specific potency of each independent lot of toxin. The availability of highly purified and well characterized research grade materials could address many of these concerns [62,63,64]. However, in some cases, the purification of substantial amounts recombinant functional BoNTs may not be possible The recently characterized BoNT/X, /Wo and /En BoNT-like ORFs presented potential safety concerns as there was no data concerning the effectiveness of currently available post-intoxication therapeutics. Preliminary data generated from the enzymatic ligation of limited amounts of the individual non-toxic recombinant domains of BoNT/X and /En indicated these toxins had no cross reactivity with antitoxins from any of the existing canonical serotypes [13,73]. While this justified the safety concerns, it also suggests developing safe, atoxic forms of these BoNTs may be useful in further characterization efforts and the development of successful countermeasures. The individual assessment of novel new subtype toxin FA (H) when expressed in a bivalent Clostridial strain B2/FA was facilitated by a KO of the B2 component of host strain [67]. However, an engineered recombinant form of the FA provided valuable information about the role of the activation loop length and characterization of the highly purified toxin [69].

The BoNTs also show promise as a DDV. The earliest efforts at using BoNTs to deliver a payload were performed using active forms of BoNT/D [75]. However, this was done as a POC and the active toxin used in this DDV used SNARE cleavage of the target cells as a metric of successful cytosolic delivery. The cargo molecules were delivered as amino-terminal fusion products that did not dissociate from the LC; but did prove BoNTs could be used to carry a functional cargo into the cytosol of the target cells. However, subsequent efforts produced atoxic DDV with LC amino terminal adaptor molecules that permitted attachment of directly conjugated or liposomal cargoes [75,76]. The data suggests the liposomal cargo system may be more flexible in terms of payload and effective delivery. The use of ABD might well be included in the subject of drug delivery. However, in this case, the drug being delivered is the functional BoNT LC-HN. Preliminary efforts were POC studies that showed the re-directed enzymes could successfully bind and internalize alternative cell types. The use of “protein stapling” techniques produced a functional BoNT/A and an /A LC-HN with a TeNT receptor binding domain [90,91,92]. Both of these molecules were found to have significantly reduced toxicity, potentially attributed to the increased size of the recombinant protein causing limited NMJ access. However, both of these stapled proteins displayed properties that suggests they may potentially influence discreet areas of the CNS without systemic toxicity. The lack of toxicity and localized CNS influence could make them a promising novel therapeutic. There have been several notable efforts in the development of engineered BoNTs designed to address pituitary hypersecretory disorders. While preliminary ex vivo and small animal studies were promising, there was negligible effect in a broad sampling of human cell lines. However, these issues may be attributed to technical limitations, the lack of a valid animal model and differences in the specificity between human and small animal receptor and SNARE target.

There have been only a limited number of studies characterizing BoNT chimeras [77,78,79,80,81]. However, the data has found the combination of serotypes provides valuable insight on the role of the individual serotype or subtype domains in the intoxication process. These studies have produced hybrids that show duration periods longer than either of the contributing parents. The combination of unique binding and proteolytic substrate specificity has shown promising applications in nociceptive pain management in pathways not influenced by the parental serotypes. Information gleaned from these novel combinatorial efforts and from future studies could also potentially allow the design of engineered BoNTs for assessment in specific applications. Finally, BoNTs designed with mutations engineered to modify substrate proteolysis have met with limited success. The information derived from mutational studies of individual domains has not translated well into full length holoproteins [82,83,85,86]. However, the partial suppression of the SNAP-25 cleavage in engineered BoNT/C1 has provided information on the specific role of syntaxin cleavage and a modified toxin that could potentially yield a novel new pharmaceutical [83]. 

There have been a substantial amount of studies describing the assessments of engineered BoNTs over the last 20 years. However, the technical advances in recombinant DNA technology and recombinant protein purification techniques have facilitated the more recent production and assessments of complex novel proteins in multistep pathways such as nociceptive pain management and regulatory developmental pathways. Many of the engineered BoNTs show tremendous application potential in the preliminary proof of concept studies. However, the data is often derived from a single study and requires independent replication and often, a more thorough characterization. The collective information from these studies strongly suggests there is great potential application of engineered BoNT derivatives to produce novel new drugs for a wide variety of therapeutic applications.

## Figures and Tables

**Table 1:** Summary of the recombinant full-length atoxic BoNT derivatives.

Serotype (Author Nomenclature)	Neutralizing Mutations	Toxicity ^a^	Reference
BoNT/C1	H^229^G,E^230^T, H^233^N	10 mcg (0.5 mg/kg)	[41]
BoNT/A1 (ciBoNT/A1)	H^223^A,E^224^A, H^227^A	50 mcg (2.5 mg/kg)	[43]
BoNT/A1 (ciBoNT/B1)	H^230^A,E^231^A, H^234^A	25 mcg (1.25 mg/kg)	[43,44]
BoNT/A1 (ciBoNT/C1)	H^229^A,E^230^A, H^233^A	25 mcg (1.25 mg/kg)	[44]
BoNT/A1 (ciBoNT/E1)	H^212^A,E^213^A, H^216^A	25 mcg (1.25 mg/kg)	[44]
BoNT/A1 (DR BoNT/A)	E^224^A/E^262^A	24 mcg (1.25 mg/kg)	[46,47,48,49,50]
BoNT/A^RYM^	R^363^A, Y^365^F	-	[51]
BoNT/A1^*ad*^	E^224^A, Y^366^A	8.9 mcg s.c. (0.445 mg/kg) 1.12 mg d.c. (0.056 mg/kg)	[53]
Cyto-012 BoNT/A	E^224^A, Y^366^A	0.63 mg/kg ^b^	[55]
BoNT/C1^*ad*^	E^238^A, H^241^G, Y^383^A	5 mg/kg	[56]
M-BoNT/A1	E^224^A, R^363^A, Y^366^F	10 mcg (0.5 mg/kg) ^c^	[59]

^a^ The value in parenthesis is an extrapolation of IP toxicity in mg/kg derived only from the published MIPLD_50_ dose and based on a 20 g mouse. ^b^ This value is a derived from an intramuscular toxicity assay in mice (IMLD_50_). ^c^ Both s.c. and d.c. forms of the M-BoNT/A1 were used in the mouse IP toxicity determination. s.c.: single chain; d.c.: di-chain.

**Table 2:** Summary of the reported drug delivery (DDV), DDV mutations, cargo attachment methods, cargoes and references.

DDV Vehicle	DDV Mutations	Cargo	Cargo Attachment Method	Reference
rBoNT/D	None	DHFR, GFP, LUC BoNT/A LC	Amino-terminal fusion	[74]
rBoNT/B1 (BoTIM/B)	E^231^A, H^234^Y	Bionylated lentivirus GFP, MTR-S25	Amino-terminal CS ^b^	[75]
rBoNT/B1 (BoTIM/B)	E^231^A, H^234^Y	BoNT/A LC ^a^, BoNT/F inhibitor ^a^, BoNT/A inhibitor ^a^	-	[76]

^a^ Represents cargo molecules encapsulated in biotinylated liposomes. ^b^ CS—core streptavidin.

**Table 3:** Summary of the chimeric/hybrid BoNTs by experimental nomenclature, domain composition, additional gene tic elements and reference.

Chimera Notation	Domain Composition	Additional Genetic Elements	Reference
BoNT/AE	/A LC-HN, /E RBD	LC-HN ELGGGGSEL linker	[77]
BoNT/EA	/E LC-HN, E RBD	LC-HC DI linker	[77,78]
BoNT/AB	/A LC-HN, /B RBD	LC-HN ELGGGGSEL linker	[79]
BoNT/BA	/B LC-HN, /A RBD	LC-HC DI linker, LVPRGS ^a^	[79]
AABB	/A LC-HN, /B RBD	LC-HN LVPRGS ^a^	[80]
A1LC/A3HC	/A1 LC-HN, /A3 RBD	None ^b^	[81]
A3LC/A1HC	/A3 LC-HN, /A1 RBD	None ^b^	[81]

^a^ Engineered thrombin cleavage site. ^b^ A 50-bp region between the LC and HC homologous between these serotypes /A1 and/A3.

**Table 4:** Summary of alternative receptor domain specific BoNT proteins by common nomenclature, LC-HN source, RBD and citation.

Nomenclature	LC-HN Source	RBD	Reference
BoNT/A LC-HN/NGF	BoNT/A	murine nerve growth factor	[87]
BoNT/A LC-HN/WGA	BoNT/A	wheat germ agglutinin	[88]
BoNT/A1 LC-HN/ECL	BoNT/A	lecithin	[89]
LcTd-SNARE-Rbd	BoNT/A	/A RBD ^a^	[90]
BiTox	BoNT/A	/A RBD ^b^	[91]
ALC-HN/CtRBD	BoNT/A	TeNT RBD	[92]
LC-HN/A-SpA-B_mut_	BoNT/A	*S. aureus* domain B composed of five highly modular immunoglobulin G (IgG) interactive domains	[93]
None Given	BoNT/A	Multiple individual domains specific to neuroendocrine cell lines	[94]
SXN101742	BoNT/D	GHRH ^c^	[95]
qGHRH-LHN/D	BoNT/D	GHRH ^c^	[96]

^a^ Native BoNT/A RBD used as POC for protein stapling method. ^b^ Independent study synonym for LcTd-SNARE-Rbd. ^c^ Same molecule with different nomenclature in independent studies.
